# Maternal care utilization and provision during the COVID-19 pandemic: Voices from minoritized pregnant and postpartum women and maternal care providers in Deep South

**DOI:** 10.1371/journal.pone.0300424

**Published:** 2024-04-29

**Authors:** Ran Zhang, Tiffany Byrd, Shan Qiao, Myriam E. Torres, Xiaoming Li, Jihong Liu

**Affiliations:** 1 Department of Health Promotion, Education and Behavior, Arnold School of Public Health, University of South Carolina, Columbia, South Carolina, United States of America; 2 Department of Epidemiology and Biostatistics, Arnold School of Public Health, University of South Carolina, Columbia, South Carolina, United States of America; National Institute of Public Health: Instituto Nacional de Salud Publica, MEXICO

## Abstract

**Background:**

The COVID-19 pandemic has significantly affected maternal care services especially for minoritized individuals, creating challenges for both service users (i.e., African American and Hispanic pregnant/postpartum women) and maternal care providers (MCPs). Guided by a socioecological framework, this study aims to investigate the experiences of African American and Hispanic pregnant and postpartum women, as well as MCPs, in accessing and providing maternal care services during the COVID-19 pandemic in the Deep South.

**Methods:**

We conducted semi-structured interviews with 19 African American women, 20 Hispanic women, and 9 MCPs between January and August 2022. Participants were recruited from Obstetrics and Gynecology clinics, pediatric clinics, and community health organizations in South Carolina, and all births took place in 2021. Interview transcripts were analyzed thematically.

**Results:**

Maternal care utilization and provision were influenced by various factors at different socioecological levels. At the intrapersonal level, women’s personal beliefs, fears, concerns, and stress related to COVID-19 had negative impacts on their experiences. Some women resorted to substance use as a coping strategy or home remedy for pregnancy-induced symptoms. At the interpersonal level, family and social networks played a crucial role in accessing care, and the discontinuation of group-based prenatal care had negative consequences. Participants reported a desire for support groups to alleviate the pressures of pregnancy and provide a platform for shared experiences. Language barriers were identified as an obstacle for Hispanic participants. Community-level impacts, such as availability and access to doulas and community health workers, provided essential information and support, but limitations in accessing doula support and implicit bias were also identified. At the institutional level, mandatory pre-admission COVID-19 testing, visitation restrictions, and reduced patient-MCP interactions were women’s common concerns. Short staffing and inadequate care due to the impact of COVID-19 on the health care workforce were reported, along with anxiety among MCPs about personal protective equipment availability. MCPs emphasized the quality of care was maintained, with changes primarily attributed to safety protocols rather than a decline in care quality.

**Conclusion:**

The pandemic has disrupted maternal care services. To overcome these issues, health facilities should integrate community resources, adopt telehealth, and develop culturally tailored education programs for pregnant and postpartum women. Supporting MCPs with resources will enhance the quality of care and address health disparities in African American and Hispanic women.

## Introduction

Maternal care utilization is critical for ensuring health and well-being of pregnant and postpartum women, especially given their vulnerability to physical and emotional changes that necessitate consistent, high-quality care [1–4]. The need for quality maternal care services is more critical for African American (AA) and Hispanic women, because they historically face systemic barriers to health care access, often leading to adverse maternal outcomes [5–7]. Studies have shown that AA and Hispanic women are less likely to receive timely and appropriate prenatal care compared to White women, with higher rates of complications during pregnancy and childbirth, and higher rates of maternal mortality and morbidity [5,8,9]. These disparities are further exacerbated in the Deep South, where structural racism and historical health inequities have perpetuated racial disparities in health care utilization [10]. In many states in the Deep South, the refusal to expand Medicaid has resulted in low-income individuals lacking access to quality and affordable health care services [7,11].

The COVID-19 pandemic has exacerbated these challenges, disproportionately disrupting maternal care services and deepening pre-existing racial and socioeconomic inequalities [12]. Maternal care service disruptions during the pandemic have ranged from canceled doctor appointments to resource shortages, mobility restrictions, and an overarching fear of COVID-19 infection [13]. Notably, during the pandemic, AA and Hispanic women have experienced a significant reduction in prenatal care visits compared to their White counterparts [14]. Barriers such as limited access to transportation and childcare support have exacerbated the decline in maternal care services utilization among these minority groups [15,16]. Moreover, the quick transition to telehealth services has not been without its own set of challenges, both for patients and maternal care providers (MCPs).

The provision of maternal care services has also been significantly impacted during the COVID-19 pandemic [17]. MCPs have had to adapt to new ways of delivering care to ensure the safety of both patients and themselves. For instance, virtual visits have been implemented as an alternative to face-to-face appointments due to personal protective equipment (PPE) shortages and infection control protocols [18]. However, this shift to virtual care has posed challenges for MCPs in effectively assessing the health of pregnant and postpartum women [19]. In addition, MCPs have reported increased workloads and reduced access to support resources, which can have a negative impact on the quality of care they provided to pregnant and postpartum women [18,20]. A study found MCPs experienced greatly increased rates of burnout during the outbreak of the pandemic, which may affect the quality of care provided to pregnant and postpartum women [21].

Adopting the socioecological model in the context of the COVID-19 pandemic allows for a comprehensive exploration of the impacts that pregnant and postpartum women experience in accessing maternal health care, as well as the strategies of MCPs in delivering essential services under unprecedented constraints [22,23]. The socioecological model assumes that individual behaviors are influenced by the context of the larger social system, emphasizing the important interactions between individuals and their environments that influence health outcomes and behaviors [24]. This framework is particularly relevant to maternal health because it allows for the identification of impacts at various levels, such as intrapersonal, interpersonal, community, and institutional, so that interventions can be tailored [25]. The interactions and mutual reinforcement of impacts at multiple levels in the socioecological model provide a comprehensive lens through which to examine the complex dynamics that affect maternal care services during the COVID-19 pandemic.

Although existing research has highlighted the enormous impacts of the COVID-19 pandemic on AA and Hispanic pregnant and postpartum women, research gaps remain in comprehensively addressing multilevel factors that contribute to interruptions in maternal care. Moreover, there is a need for research that examines the perspectives and experiences of MCPs who serve AA and Hispanic populations. Guided by a socioecological framework [24,26], this study aims to investigate the experiences of AA and Hispanic pregnant and postpartum women, as well as MCPs, in accessing or providing maternal care services during the COVID-19 pandemic in the Deep South.

## Methods

### Study design and participants

We adopted qualitative interviews to explore AA and Hispanic women’s experience of seeking maternal care services during the COVID-19 pandemic. Semi-structured interviews were chosen due to their flexibility, which enabled participants to share their experiences and perspectives in their own words while still guiding the conversation through a set of pre-determined questions. Purposive sampling was utilized to recruit postpartum women and MCPs. Participants were approached through local Obstetrics and Gynecology (OBGYN) clinics, pediatric clinics, and community health organizations in South Carolina. These sites were selected due to their engagement with low-income AA and Hispanic pregnant and postpartum women. Eligible postpartum women were aged 18 years or older, self-identified as AA or Hispanic, had given birth in 2021, and living in South Carolina. In total, we conducted 48 semi-structured interviews with 19 AA women, 20 Hispanic women, and 9 MCPs between January and August 2022. This study received approval from the University of South Carolina Institutional Review Board (Pro00115169).

### Data collection

Semi-structured interviews for postpartum women were facilitated by two researchers, conducted via Zoom, phone, or in-person. Interviews with Hispanic participants were conducted in Spanish. The interviews guide for postpartum women (S1 Table) covered topics such as 1) mothers’ perceptions toward their health care providers and institutions for perinatal care, 2) experience with prenatal and postpartum care, 3) stressors experienced during the COVID-19 pandemic, 4) challenges in health care seeking (e.g., appointments and clinic visits), especially related to structural factors (e.g., racism and discrimination), and 5) mothers’ needs and recommendations for future health care. The interview guide for MCPs (S2 Table) aimed to capture their perspectives on 1) stressors and challenges of their clients, 2) clients’ mental health conditions, 3) impacts of COVID-19 on their care provision, and 4) health disparities caused by structural factors. Researchers provided an explanation of the research’s purpose, and all participants provided written informed consent before participation. Participation was voluntary, and participants could withdraw at any time. Upon completion of the interviews, each participant received a $50 gift card. Interviews were audio-recorded with consent, lasting 50 minutes each.

### Data analysis

All interviews were transcribed verbatim. Interviews conducted in Spanish were translated into English by a bilingual researcher (MT). Microsoft Excel was used to manage the data. The anonymized transcripts were independently analyzed by two researchers (TB and RZ) using the six steps of inductive thematic analysis (i.e., data familiarization, initial coding, searching for themes, reviewing themes, and finally defining and naming themes) [27]. After reviewing the transcripts, both researchers independently applied descriptive codes to the data and condensed and categorized the codes to identify themes. Overarching themes regarding intrapersonal, interpersonal, community, and institutional level impacts were created through discussion between the researchers (TB, RZ, and SQ). Any coding discrepancies during data analysis were resolved by both researchers after revisiting the transcripts and discussing with all authors. Codes and themes were revised iteratively to ensure accuracy and meaning.

## Results

### Demographic characteristics

Every postpartum woman who participated in the study gave birth in 2021 at a hospital in SC. The AA participants, aged from 21 to 42 years, all had at least a high school diploma or general educational development (GED), with 21% achieving a master’s degree. A majority, 74% of those AA participants were not first-time mothers and had previously engaged with maternal care services, even before and during the COVID-19 pandemic. Regarding insurance coverage, 58% of AA participants’ childbirth expenses were covered by Medicaid, 37% by private insurance, and 5% by themselves. Most of these AA participants lived in Richland (32%) and Spartanburg (26%) Counties. The Hispanic participants, aged between 19 and 41, included 50% without a high school diploma or GED and 15% with a bachelor’s degree as their highest education attainment. All the Hispanic participants were immigrants, with 75% having had previous childbirths. In terms of insurance coverage, 70% were covered by Emergency Medicaid, 5% by Medicaid, 5% by a special program, and 20% by themselves. Most Hispanic participants resided in Richland (45%) and Lexington (45%) Counties. The interviewed MCPs had different occupations, including obstetrician and gynecologist (n = 4), certified nurse-midwife (n = 2), nurse practitioner (n = 2), and nurse navigator (n = 1), consisting of eight females and one male. Five MCPs worked at clinics primarily serving AA patients (50–80%), and three MCPs worked at clinics that served more balanced mix of AA and Hispanic patient populations, each group comprising 20–40% of the patient population (Table 1).

**Table 1:** Sociodemographic characteristics of the participants.

***Characteristic***	***African American Participants (%)*** ***N = 19***	***Hispanic Participants (%)*** ***N = 20***
**Age Range**		
18–25	3 (16%)	5 (25%)
26–35	12 (63%)	8 (40%)
36–50	4 (21%)	7 (35%)
**Education**		
Less than high school diploma	0	10 (50%)
High school diploma or general educational development	9 (47%)	6 (30%)
Associate degree	1 (5%)	0
Bachelor degree	5 (26%)	3 (15%)
Master degree	4 (21%)	0
No education	0	1 (5%)
**Marital Status**		
Single	2 (11%)	6 (30%)
Married	9 (47%)	6 (30%)
Living with unmarried partner	8 (42%)	8 (40%)
**Number of Children**		
1	5 (26%)	5 (25%)
2	8 (42%)	3 (15%)
3	4 (21%)	6 (30%)
4	2 (11%)	3 (15%)
>4	0	3 (15%)
**Insurance Coverage**		
Medicaid	11 (58%)	1 (5%)
Private insurance	7 (37%)	0
Self-pay	1 (5%)	4 (20%)
Emergency Medicaid	0	14 (70%)
Other/program	0	1 (5%)
**Place of Birth**		
USA	19 (100%)	0
Colombia	0	4 (20%)
Mexico	0	4 (20%)
Guatemala	0	6 (30%)
Honduras	0	4 (20%)
Costa Rica	0	1 (5%)
Unknown	0	1 (5%)
**County of Residence**		
Darlington	1 (5%)	0
Spartanburg	5 (26%)	0
Colleton	3 (16%)	0
Beaufort	1 (5%)	0
Richland	6 (32%)	9 (45%)
Sumter	2 (11%)	0
Lexington	1 (5%)	9 (45%)
Berkeley	0	1 (5%)
Unknown	0	1 (5%)
***Characteristic***	***Maternal Care Provider*** ***N = 9***	
**Age**		
26–35	1	
36–45	3	
46–55	3	
56–65	2	
**Tenure at Current Practice/Hospital**		
<1 year	2	
1–5 years	4	
6–10 years	3	
**Job Title/Specialty Area**		
Obstetrician and Gynecologist	4	
Certified Nurse-Midwife	2	
Nurse Practitioner	2	
Nurse Navigator	1	

Note: Percentages do not add up to 100% due to rounding.

Our study investigated experiences of maternal care utilization and quality among AA and Hispanic women during the COVID-19 pandemic in SC. We interpreted these experiences through the socioecological model [25,28], which helped us categorize them across four levels: intrapersonal, interpersonal, community, and institutional (Table 2). The narratives provided by participants at each socioecological level highlight their maternal care experiences and the quality of services they received during the COVID-19 pandemic in maternal health clinics and hospital settings.

**Table 2:** Key themes.

Main Themes	Sub-Themes
Intrapersonal level: fears and stress during the pandemic	Personal beliefs and discomfort with certain MCPs, influenced women’s maternal care decisions during the pandemic.
	Women experienced heightened stress due to the multifaceted roles they juggled during the pandemic.
	Increased substance use in patients as a coping mechanism, MCPs suggest safer symptom management alternatives.
Interpersonal level: family and social network	Spouses infected with COVID-19 complicated childcare and visits.
	Families provided key support during medical appointments.
	Community groups were desired for shared pregnancy experiences.
	Language barriers affected Hispanic participants.
Community level: doulas and community health workers	Doulas and community health workers provided vital support to women during childbirth in COVID-19.
	Challenges included mask-related communication barriers and bias against black doulas.
	A demand for more accessible doula services, especially among communities of color.
Institutional level: new policy and regulations implemented in health facilities, and uncertainty associated with changing policies	Institutional change: Mandatory pre-admission testing and visitation rules.
	Visitation concerns: Restrictions made many women feel violated, leading some to explore homebirth options.
	Testing debate: Mandatory COVID-19 testing affected patient-MCP relationships and care promptness.
	Staffing issues: Pandemic-related staffing challenges compromised care due to MCP shortages and inflexible nursing.
	Application challenges: Adapting general COVID-19 insights to pregnancy scenarios posed immediate obstacles for medical facilities.

### Intrapersonal level: Fears and stress during the pandemic

During the COVID-19 pandemic, women’s personal beliefs, fears, concerns about accessing care, and the impact of the pandemic on stress levels influenced their decisions and experiences during pregnancy. One participant expressed discomfort with certain MCPs and stated that she would request a provider change if she knew that particular MCP was scheduled. This discomfort had a negative impact on her mindset and diminished her overall experience when attending appointments. Examples of quotations to meet the theme of *intrapersonal level*: *fears and stress during the pandemic* include:

“*If I saw her name [MCP’s name]*, *I’d be like*, *y’all can’t change it*? *And most of the time*, *she was the only one available because everybody else had surgery*. *So that didn’t make me not want to go*. *But it didn’t put me in the right mindset before I showed up*. *It wasn’t like I was excited*, *but it’s kind of hard to be excited when she’s gonna ruin it for me*.*”* (AA 7)

Concerns about the process of accessing care and the potential risk of getting COVID-19 themselves and their babies were also reported. Some participants expressed their concerns and stress about attending doctor’s appointments during the pandemic and emphasized the importance of staying safe. MCPs highlighted the hesitation to come in for necessary care due to the fear of exposure to COVID-19. Examples of quotations to meet the theme of *intrapersonal level*: *fears and stress during the pandemic* include:

“*I am worried about that*, *when I go to these doctor’s appointments*, *am I gonna bring something back to her*?… *I was very cautious*.*”* (AA 13)

“*But all in all*, *it was stressful too*, *like going to the doctor with COVID going on*, *and having to wear a mask*, *and you’re just trying to stay safe*.*”* (AA 15)

*“It’s also part of the patients as well not wanting to come in because they don’t want to be exposed to COVID either*, *that maybe people don’t come in soon enough for things that they needed to come in for*, *like high blood pressure issues*, *and just various things*.*”* (MCP 3)

Some women sought guidance from MCPs regarding their concerns about COVID-19. While some participants felt supported and helped by their interactions with MCPs, others perceived their experiences as inefficient and lacking in education. Examples of quotations to meet the theme of *intrapersonal level*: *fears and stress during the pandemic* include:

“*The COVID had me stressed out ’cause I didn’t know how it would affect a pregnant person*, *and so I talked to my doctor*, *and they advised me to get the vaccines and everything*.*”* (AA 19)

*"When we got COVID*, *I was extra scared because of not knowing how it affected her [the baby]*. *Our doctor*, *she really*, *I’m not gonna say brushed me off*, *but I call her to tell her that*, *I was just diagnosed with COVID-19*. *What do I do*? *And her exact words were*: *‘Nothing*. *We don’t treat pregnant mothers differently than we do any other person that has COVID’*. *And that kind of scared me because I’m just like*, *well*, *how would COVID affect my baby*? *It was no education behind nothing*.*”* (AA 4)

Living and managing care during the pandemic served as a source of stress for many women. Participants, regardless of their feelings about pregnancy, reported increased stress due to the pandemic’s impact on their daily lives. This type of stress resulted from the multiple roles they took on during the pandemic, including being a full-time mom, a full-time worker, and a wife. Examples of quotations to meet the theme of *intrapersonal level*: *fears and stress during the pandemic* include:

“*During pregnancy*, *one is very sensitive and if you add all the issues with the pandemic*, *everything is complicated*.*”* (Hispanic 6)

“*Even if you’re happy about being pregnant*, *whereas some people are still ambivalent*, *it’s still stressful*.*”* (MCP 5)

"*Yes*, *very stressed*. *Very stressful*. *Being a full-time mom*, *being a full-time worker*, *being a wife*, *during a pandemic is not easy*.*”* (AA 4)

MCPs also reported an increase in patients disclosing substance use as a coping strategy or home remedy for pregnancy-induced symptoms, such as morning sickness. Marijuana use was frequently mentioned by their patients, with some freely admitting to using it during pregnancy. Some MCPs noted an increase in patients reporting marijuana use to help them relax and manage nausea. Moreover, MCPs emphasized the importance of discussing safer alternatives for managing pregnancy-related symptoms. Examples of quotations to meet the theme of *intrapersonal level*: *fears and stress during the pandemic* include:

"*I do have a lot of people that just freely admit to smoking marijuana*, *even pregnant*… *I’m like*, *‘let’s talk about that’*.*”* (MCP 5)

“… *probably marijuana use*. *We ask in terms of tobacco and alcohol*. *We have very few patients that will admit to alcohol and tobacco*. *A lot of our patients*, *it’s marijuana and [I’ve] probably seen an increase in that just them saying*, *it helps me relax*.*”* (MCP 2)

"*They said to me*… *‘I’ve done that for the nausea in pregnancy’*. *[It] was one of the things that they’ve always said is*,… *‘I find that*, *that helps when I’m nauseous’*. *I’m like*, *well*, *there are safer things that you can do for nausea than that*.*”* (MCP 1)

“*I’m like*, *are you still smoking marijuana*? *[patient says] ‘Yeah’*, *I’m just like*, *Okay*, *well*, *we’ll do the third trimester drug screen*. *They’re like*, *okay*, *but they don’t seem to be bothered by that*. *But they disclose it*. *I’m very impressed that patients disclose more of that now since COVID*.*”* (MCP 1)

### Interpersonal level: Family and social network

Participants’ responses demonstrated a strong awareness of interpersonal level influences on maternal care utilization and treatment choices. These impacts included changes in family, friends, and other social networks as a result of viral transmission and pandemic control. Some MCPs mentioned situations where their patients’ spouses were infected by COVID-19 and needed to be isolated, creating challenges in caring for newborns and managing other children. Moreover, some women stated that concerns about COVID-19 exposure affected decisions regarding accompanying infants to doctor appointments. Examples of quotations to meet the theme of *interpersonal level*: *family and social network* include:

“*If a spouse gets COVID*, *that’s happened a number of times the spouse got COVID and had to isolate*. *So now you’ve got a new baby*. *Her spouse is in the house*, *can’t help you at all*. *There’s other little children*. *And now you’re taking care of a new baby*, *and you just have to see*, *it’s just a mess*.*”* (MCP 5)

“*I didn’t even bring [infant’s name] with me [to doctor appointment]*, *even though they told me over the phone*, *I could*. *But I didn’t bring her for personal reasons*. *Because of COVID*. *’Cause I didn’t want her to be exposed and be around her sister*. *But I think I think maybe if we weren’t in a pandemic*, *she probably would have been with me*.*”* (AA 13)

Participants indicated that social networks, particularly family members, played a crucial role in facilitating access to maternal care services, with some participants highlighting instances where relatives provided childcare support. Examples of quotations to meet the theme of *interpersonal level*: *family and social network* include:

“*If I had a doctor’s appointment and dad wasn’t around*, *she [participant’s mother] would pick us up and take us*.*”* (AA 17)

“*My mom had him the whole time I was in the hospital*.*”* (AA 18)

“*I guess yeah*, *sometimes his great grandma would keep him*, *but it would be like if I had an appointment*.*”* (AA 2)

“*Yes*, *when I had to go to appointments*, *a friend helped me or pick up my girl from school*.*”* (Hispanic 6)

Several participants, especially those in the AA group, emphasized the importance of having access to resources that allowed them to learn with and from other women who were also going through pregnancy and navigating new motherhood. They expressed the desire for sister groups, community groups, and other support groups to alleviate the pressures of pregnancy and provide a platform for shared experiences. Examples of quotations to meet the theme of *interpersonal level*: *family and social network* include:

“… *sister groups most definitely*… *You think you’re going through a lot with your pregnancy*, *and [to know] there’s someone out there shares the same fears and things*, *and I feel like um sister groups like community groups and things of that sort can kind of lay off the pressure of pregnancy*. *Like you’re not in this by yourself*.*”* (AA 13)

“*Like even a support group for*, *even for people who just need someone to talk to*.*”* (AA 3)

“***…*** *probably like how the [name of program] that they have there in Columbia*. *I think they should probably have something like that with um parents here too*. *At least somebody to walk me through the entire pregnancy*… *just ‘cause you get WIC [Women*, *Infants and Children Nutrition program]*, *WIC is not enough*.*”* (AA 16)

However, women and MCPs agreed that the discontinuation of group-based prenatal care, such as Centering Pregnancy groups, due to the pandemic was detrimental to maternal care. MCPs emphasized the benefits of group settings, where women could learn form and support each other, and expressed the need for community efforts to resume such programs. Examples of quotations to meet the theme of *interpersonal level*: *family and social network* include:

“*I do know*, *at [hospital name] as I’m sure that you’ve heard*, *the centering was shut down*, *and it is having a lot of trouble being picked back up*.*”* (MCP 9]

“*Centering Pregnancy model is really a great model*, *because*…*you don’t know what you don’t know*. *And so*, *if you’ve got 20 women in a room*, *and they’re doing this*, *they a lot of times will feel power in having people like them*, *they’re pregnant*, *or they’re all African American*, *or they’re Latina*, *or they’re a combination*, *but there’s more pregnant people in the room*, *than there are doctors*, *or nurses or whatever*. *So*, *that dynamic*, *it shifts*, *it really does*. *And they ask questions*, *and one of them will ask [a] question that the other one didn’t even know to ask*, *and she’s like*, *‘Oh’*, *and they learn from each other*. *And I think*, *I think that it’s really powerful*, *and COVID just kind of destroyed that in so many ways that for those centering groups*.*” (MCP 5)*

*“I think I would like to see more community efforts to kind of recover that*, *like centering that we had*, *like from all the things I learned about centering*, *it sounded great*. *Like it was improving outcomes*. *It was improving like the mom’s mental health*, *and then they started to have like*, *centering for parenting*.*”* (MCP 8)

Language barriers were identified as a significant obstacle for Hispanic participants in accessing group-based prenatal care, with a call for tailored classes for Spanish-speaking women to address this barrier. Examples of quotations to meet the theme of *interpersonal level*: *family and social network* include:

“*But they’re not getting a two-hour diabetes and pregnancy class*, *they’re not getting group education*. *And I think there’s benefits of the group setting as well and knowing that you’re not alone*, *and they*, *they’re not getting the same education and care simply because of the fact that there’s the language barrier*. *So I would like to see classes for the Spanish speaking women*.*”* (MCP 9)

### Community level: Doulas and community health workers

In supporting women who give birth during COVID-19, community support and resources, such as doulas and community health workers, played a crucial role in providing care and filling gaps in when women were hesitant or unable to visit clinics. Community-based support systems can effectively address the informational and emotional needs of women during challenging times. Many participants emphasized the value of community health workers and doulas in providing essential information and support. Women’s experience with community health workers was positive, as they kept them informed and involved their family members when necessary. One participant mentioned that doulas can provide personalized and compassionate care that goes beyond what is typically available in a hospital setting. Examples of quotations to meet the theme of *community level*: *doulas and community health workers* include:

“*’Cause they kept me straight*. *They were like*, *‘Listen*, *if you don’t do this*, *this is gonna happen*. *If you do this*, *this’ll happen’*. *They were straight up forward*. *Letting me know everything*, *always gave me the information I needed*. *Um*, *if they couldn’t talk to me*, *my mom and fiancé were always on call*. *So*, *they would let them know everything*. *So*, *they were on top of everything*.*” (AA 19*)

"*Her care was amazing*. *Through the third trimester and throughout my labor*, *I was an anxiety wreck ’cause she was coming a month early*… *I’ll never forget*, *I was crying*. *I was like*, *I’ve never even got my hair braided*. *’Cause was supposed to go get my hair braided the next day*… *So I was just crying like mid-contraction*. *I was like*, *I didn’t even get my hair braided*. *So she braided my hair*. *She braided my hair in like two little French braids*. *And that just made me just feel just*, *it was like a detail like that*, *that I know*, *I wouldn’t have gotten at a hospital*. *She was like*, *it’s okay*, *I’ll braid your hair*. *So she just braided my hair*. *So that way when I gave birth*, *my hair wasn’t going everywhere looking crazy*. *It was immaculate care*.*”* (AA 6)

*“She supported me a lot*. *With visits and by phone*. *Each time she visited me*, *she helped me because everything was more difficult due to the pandemic*. *She facilitated everything for me*.*”* (Hispanic 20)

“*They gave me fliers with all the care*. *The reasons why we needed to go back to the hospital*.*”* (Hispanic 19)

However, some participants faced obstacles and limitations in accessing doula support due to the pandemic. Several participants shared the challenges of interpreting social cues and limited visibility during childbirth because everyone was wearing masks. Moreover, a MCP pointed out the issue of implicit bias, particularly towards Black doulas, resulting their exclusion from providing support. Examples of quotations to meet the theme of *community level*: *doulas and community health workers* include:

“*Everybody was wearing masks*. *So I couldn’t really gauge the excitement*. *I guess that kind of threw me ‘cause*, *my doula was in a mask*. *Everybody was in a mask*. *The only people that weren’t in masks were obviously me and my husband*. *So I think the*, *the social cues of*, *is she coming*?… *I can’t really see what’s happening right now*.*”* (AA 6)

*“Um also came to find out after working with a group of doulas that there were doulas that were being turned away and the doulas that were being turned away*, *were black doulas*. *So*, *we also have the implicit bias going on*, *because people are making decisions about who can be there and who can’t be there*. *And that didn’t used to be an issue when whoever could be present*.*”* (MCP 3)

Some women expressed a desire for increased access to doula services and the need for programs that can provide support and disseminate vital information, especially among communities of color. A Hispanic woman expressed disappointment in not being offered doula services and highlighted the lack of information provided to her. Some AA women mentioned the misconception that doula services were expensive and inaccessible. Examples of quotations to meet the theme of *community level*: *doulas and community health workers* include:

“*I would have liked to have been offered doula services*. *I know what a doula is and how that service could have benefitted me because of this support for the mother*, *especially for me that I didn’t have any support during the delivery with me*. *I know moms from other races that they get offered doula services and to me they didn’t offer it and didn’t even tell me they had that service*.*”* (Hispanic 3)

"*As a Black woman*, *giving birth in the US and*… *giving birth at home*, *I think a program like that will be very beneficial*. *If people knew that these services are not expensive*. *I went into the impression when I started this*, *that this was gonna be so expensive*, *I could never afford this*, *I would have to just settle for the hospital*. *Because that’s just all we were taught*. *And that’s all I knew*. *Nobody told me anything*… *And we just assume not trying to be funny*, *but only white people can afford them*. *And that’s not true at all*.*”* (AA 6)

### Institutional level: New policy and regulations implemented in health facilities, and uncertainty associated with changing policies

Institutional factors had a significant influence on women’s ability to acquire the desired maternal care experience, as highly reported by participants. Changes in COVID-19-related hospital and clinic policies and practices had a notable effect on women’s maternal care experience. Mandatory pre-admission COVID-19 testing, visitation restrictions, and discouragement of lengthy one-on-one patient-MCP interactions were commonly mentioned concerns by both women and MCPs. Examples of quotations to meet the theme of *institutional level*: *new policy and regulations implemented in health facilities*, *and uncertainty associated with changing policies* include:

“*But the policy changes that*, *that wouldn’t allow partners to come or any flexibility*… *or the screening practices and*… *all of the questions around who might be positive or exposed*, *it turned a lot of women away from appointments and care*, *like if they showed up with their children*, *they were turned away*, *or if they had a temp*, *or if they had a sniffle*, *they might be turned away and not cared for*.*”* (MCP 9)

*“[patients would ask] why do I have to get a babysitter to come to prenatal care*, *when I used to be able to bring my kids with me to the visits*? *And a lot of them can’t afford to do that*. *So*, *we see a lot of missed visits because of that*, *they didn’t have a babysitter*.*”* (MCP 1)

“*Yes*, *I was panicked about not knowing about the pandemic and that we couldn’t do the prenatal visits normally*. *I had to go in by myself because they didn’t allow my husband to be with me*.*”* (Hispanic 20)

“*The only thing that was different was not having my husband come to any of the appointments*. *So*, *um I went into everything alone*. *Like honestly*, *the only appointment he went to was when I delivered and that that was it*. *That’s the only appointment he was able to come to so just feeling I wasn’t*, *I wouldn’t say I was scared because I’ve been through many doctors’ appointments*, *but it was just that extra sense of comfort when he was there with my*, *our son*. *I just wasn’t able to have him there with our daughter which was*, *it was scary man*.*”* (AA 4)

Visitation restrictions resulted in some participants feeling that their rights were violated, preventing them from having an accompanying person during childbirth. This led some participants to explore alternative options, such as homebirth. Examples of quotations to meet the theme of *institutional level*: *new policy and regulations implemented in health facilities*, *and uncertainty associated with changing policies* include:

“*When I found out I was pregnant*, *they weren’t allowing anybody in the birthing room besides the mother*, *the nurse and the doctor*. *So*, *no family*, *no anything*. *And that made me anxious because I’m a first-time mom*, *I’ve never given birth before*. *I was like*, *of course I want my husband there*, *at least my mom*, *but I would take my husband*, *but it was in the beginning my gynecologist was like*, *No*, *you can’t have anybody*. *And that just immediately turned me off*. *So*, *I started looking at other things*.*”* (AA 6)

“*I felt like my rights were being*, *excuse me*, *my rights were being violated as a patient because you have the right to moral support*. *So*, *I just felt like I understood this was for safety purposes*, *but it was a little bit too strict*, *just not having that one person*.*”* (AA 11)

Participants had mixed reviews on mandatory COVID-19 testing requirements. While the testing requirements aimed to ensure safety, they also affected MCPs’ ability to provide timely care and impacted the patient-MCP relationship. Examples of quotations to meet the theme of *institutional level*: *new policy and regulations implemented in health facilities*, *and uncertainty associated with changing policies* include:

“*We used to not obsess over patients*, *they’d get a cold*, *or they’d get a sniffle*, *or they’d get a flu*, *we would just provide care to them and take care of them*. *Now we’re like*, *Oh*, *you gotta go get a COVID test before we can even see you*. *I think it’s really affected how we provide care to patients*. *Everything is like everything’s rule out COVID before you see the patient*.*”* (MCP 1)

“*I was concerned going in*, *’cause they have to COVID test you to go in*, *which they didn’t COVID test my husband*, *which was so stupid*. *I was like*, *that doesn’t make any sense to me*. *But anyway*, *so I had to get a COVID test*.*”* (AA 10)

“… *that visitation policy and have to be subjected to the COVID testing*. *That was very*, *it was a straight violation of me*. *I just didn’t like it*. *The mandatory testing*.*”* (AA 11)

Limited physical touch and interaction due to COVID-19 prevention practices were concerning to both MCPs and women. This lack of close support and reduced physical contact compromised the quality of care and support provided during pregnancy and childbirth. Examples of quotations to meet the theme of *institutional level*: *new policy and regulations implemented in health facilities*, *and uncertainty associated with changing policies* include:

“*I do know that when I was a nurse*, *and supporting a patient with an epidural*, *not every nurse*, *but I feel like the good nurses really supported their patients*, *you touch them*, *you’re close to them*. *They have to lean up around you and hold on to you to get their back in the right position*. *But if you’re all masked up and afraid to COVID*, *you’re just not touching people very much*, *you’re not putting your hands on them*. *And getting right there with them to provide that hands on support and care*.*”* (MCP 9)

"*Um*, *I honestly think the thing that led to the quality of care issues*, *and I do think in some ways the quality was diminished*… *I think that that was probably due to the implicit bias that we had*. *And the fear that providers and nursing had*, *that they were going to get the virus*. *So we*, *I’m gonna use we as me included*, *even though I tried not to do this*, *but we would do everything we could not to see the patient if she was in that 10 day window of having COVID*. *And I think sometimes that was done*. *Those decisions were made out of concern*, *slash fear*, *for [the] provider team more than it was for the patient themselves*. *And we probably didn’t see some patients that we should have seen or that we would normally would have seen*.*”* (MCP 7)

"*And then also*, *if there’s a concern that somebody’s positive*, *or they are positive*, *they’re getting less one on one care*, *because you have to get in your full garb and dressed up so they’re not getting the support that they were getting before*. *So now*…*they don’t have their labor support people with them and now they don’t have their nurses with them either*. *So*, *I think that’s a huge difference*.*”* (MCP 3)

*“So now that covid is out*, *whether you vaccinated or not*. *I feel like they do get a little nervous closed up in there*. *So they’re not taking the time that they usually do*, *or they get you in and out*.*”* (AA 7)

Both women and MCPs commented on the impact of COVID-19 on the hospital or clinic workforce, resulting in short staffing and delayed or inadequate care. The increased number of MCPs quitting due to the pandemic further exacerbated the staffing challenges and affected the provision of care. Examples of quotations to meet the theme of *institutional level*: *new policy and regulations implemented in health facilities*, *and uncertainty associated with changing policies* include:

"… *and then it affects staffing*, *when patients come into the office sick*, *we have staff members that then get sick with COVID*. *And then they’re out*, *which then affects how much care we can provide*.” (MCP 1)

“*Hmm*. *I mean*, *probably because I think due to COVID I don’t know if hospitals were always short staffed in that manner*. *But I know that there was a boom of healthcare workers quitting after COVID happened*. *So they were extremely short staffed*. *It did play a part in*… *me not getting the aftercare that I was supposed to get after delivery*.*”* (AA 18)

*"There’s less tolerance*, *I guess of*, *of people who are wanting things*, *like for birth*, *a lot of people have their birth plans*, *and they’d like to have things a certain way*. *And I think the nursing staff is a little shorter than they used to be*. *And part of that is the burnout that’s happening for them*. *So*, *they’re not as flexible and*, *and that definitely affects patient care*.*”* (MCP 3)

“*And then in the hospital*… *when we started to recognize that pregnant patients fared very poorly with COVID*… *trying to a lot of times*, *doing it on the fly*, *trying to extrapolate information from other spheres of medicine to apply to a pregnant patient and breastfeeding patients*, *and so forth*, *that that was challenging*. *There’s a lot of on your feet thinking and extrapolating from other parts of medicine*.*”* (MCP 5)

Challenges in the supply chain of PPE hindered the provision of a secure healthcare environment for pregnant and postpartum women. In addition, insufficient PPE availability resulted MCPs experiencing anxiety about rationing and efficiently using essential PPE. Examples of quotations to meet the theme of *institutional level*: *new policy and regulations implemented in health facilities*, *and uncertainty associated with changing policies* include:

“*Cause you had to take everything off*. *At the time PPE was not as*, *it was scarce*, *I mean*, *you were kind of worried like*, *am I gonna have all the supplies*? *I need to actually care for somebody without making myself sick*.” (MCP 4)

*“There was the whole thing about like*, *we had a shortage of PPE for a while*… *And then it was like*, *they kind of kept relaxing their requirements when we didn’t have enough PPE and saying*, *like*, *‘oh*, *you actually can wear the same N95 all day’*… *they only said that*, *because we didn’t have enough*.*”* (MCP 8)

*“At the very beginning with donning and doffing with PPE that was initially supervised*, *there was like always a watcher to watch and make sure you did it properly*. *So*, *that of course changed when we didn’t have enough people to do that*. *And then I think it’s masking is obviously the biggest one*, *having really strict rules about masking*, *who is masked what having to wear a mask all the time*, *and that is constantly under flux*.*”* (MCP 6)

Some MCPs mentioned the implementation of telehealth appointments aimed to ensure continued care for patients with COVID-19 and limit in-clinic services during the early stage of the pandemic. However, telehealth posed challenges in maintaining the personal contact and physical assessment needed for comprehensive care. The technology and tools to obtain telehealth visits varied, leading to inequities in care delivery. Despite the challenges, the increased use of telehealth during the pandemic highlighted its benefits for health care service delivery, offering convenient options for short interactions and overcoming geographical barriers. Examples of quotations to meet the theme of *institutional level*: *new policy and regulations implemented in health facilities*, *and uncertainty associated with changing policies* include:

“*It did open up the opportunities for virtual visits*… *But it took away that personal touch*, *and the ability*, *particularly in pregnancy*, *when you really need to lay your hands on a mom’s belly*, *listen to the heartbeat*, *look at the patient*. *You can only go so far with a virtual visit*. *So*, *it decreased that personal touch and*, *and I think decreased our ability to truly physically assess the patient as well as we would like to in a normal situation*.*”* (MCP 7)

“*A lot of the technology that we had just wasn’t accessible to those patients*. *So it’s all well and good to say we’ll do a video visit and have you check your own like blood sugars at home*, *but if you can’t access a glucometer or glucose strips because you can’t afford them*, *or you have spotty Wi-Fi or you live somewhere rural where you don’t have Wi-Fi*, *then that’s not really a possibility*. *So*, *I just felt like they didn’t have the same access to tools that other people were using to try and overcome pandemic stuff*.*”* (MCP 6)

“*One of the good things that has come out of COVID is the expectation that people will be able to use telemedicine in some capacity*, *or that we will have some sort of like*, *drive through some sort of component of healthcare*. *And that has been actually very good because those one-on-one interactions for short periods of times actually do serve a great purpose*… *if you need to have a telehealth visit*, *whereas before it was a complete novelty*.*”* (MCP 5)

During the COVID-19 pandemic, the rapidly changing policies created an environment of uncertainty and complexity for both women and MCPs. MCPs expressed the challenges of providing clear expectations to patients, and they faced difficulty in navigating patients. Most MCPs acknowledged that patients might feel bothered by the changing policies. However, one MCP emphasized that the overall care provided had not decreased or changed, indicating that the changes were primarily related to safety protocols rather than decline in the quality of care. Several Hispanic women expressed ambiguous or poorly communicated policies led to confusion. Examples of quotations to meet the theme of *institutional level*: *new policy and regulations implemented in health facilities*, *and uncertainty associated with changing policies* include:

"*I think one of the challenges*, *is*… *the changing policies*, *when patients ask*,… *when I go to have my baby*, *how many people can come*? *Or can it be two people in the room now*? *And or can they switch out*, *and I’m like*, *it changes so much that I don’t know how to*, *to counsel them to give them clear expectations*.*”* (MCP 9)

"I *think patients are more bothered by it*. *In terms of they feel like we’re treating them differently*. *And I mean I guess we are*, *but it’s not their fault*. *I mean it’s not them per se*.… *I think patients may say that*… *because it may not be as personal when you have a gown and a mask and a face shield and gloves and all that on*, *but I don’t think the overall care has decreased or changed*.*”* (MCP 2)

“*COVID affected because now there are too many rules*. *Everybody has to wear a face mask and I couldn’t go to the appointment with someone else*. *I had a different idea of how this was going to go*.*”* (Hispanic 17)

“*It was very confusing*. *They took a long time to get appointments*. *They didn’t allow anyone to be with you*. *When one has a complicated pregnancy*, *one needs support*.*”* (Hispanic 2)

## Discussion

This study applied the socioecological framework to understand the multifaceted impacts of the COVID-19 pandemic on maternal care utilization among AA and Hispanic pregnant and postpartum women in the Deep South. By focusing on a predominantly low socioeconomic and underserved population, including many immigrants, it illuminates the specific challenges these groups face in accessing maternal care. Our comprehensive approach, integrating perspectives from both patients and MCPs, reveals the multifaceted and layered influences on maternal care utilization. This nuanced analysis extends beyond individual behaviors to include interpersonal, community, and institutional dimensions, providing valuable insights into targeted interventions that could mitigate the disparities in maternal health care. The study not only deepens the understanding of how pandemic-related stressors and systemic barriers impact AA and Hispanic women but also offers a model for future research and policy interventions aimed at improving maternal health equity. Our findings contribute to existing research highlighting personal struggles and the systemic barriers that affect their maternal care experiences during the pandemic.

The COVID-19 pandemic has caused unprecedented stressors for AA and Hispanic pregnant and postpartum women, significantly affecting their experience of seeking maternal care. Notably, our findings reveal a heightened fear of virus exposure as a critical barrier to seeking maternal care, a concern distinctly amplified during the pandemic. At the intrapersonal level, we found that personal beliefs and fears related to COVID-19 risk significantly impact decision-making for seeking maternal care services. This finding emphasizes the role of risk perceptions in influencing health care seeking behaviors during outbreaks [29,30]. Moreover, we identified stress associated with managing maternal care amidst the pandemic as a significant concern for many women. MCPs reported an increase in women disclosing substance use to cope with stress or as a home remedy for pregnancy-induced symptoms, indicating disparities in access to mental health support services and the need for targeted interventions during the challenging time [31,32].

At the interpersonal level, social networks and support systems played a critical role in determining when pregnant and postpartum women access maternal care services during these challenging times. The multiple roles women took on during the pandemic caused much stress, and their social support networks could help them reduce stress and address pregnancy confusion [33]. In our study, many participants emphasized the importance of resources that allowed them to learn from and share experiences with other women going through pregnancy and navigating new motherhood. The discontinuation of group-based prenatal care due to the pandemic was seen as detrimental to maternal care, especially by MCPs, who felt that centering groups were effective in maximizing their time while providing social support for participants. However, we also identified language barriers as a major limitation to accessing group-based prenatal care among Hispanic participants, which contributes to the unique challenges among Hispanic women to care, such as language barriers and a lack of cultural competency on the part of health care personnel [34,35]. This finding emphasizes the need for culturally sensitive and linguistically appropriate care to address health disparities among diverse populations.

Community-level support, including the presence of doulas and community health workers, has emerged as crucial sources of support for pregnant women during the pandemic, providing comfort and guidance during uncertain times [36]. These community-based support systems effectively address the informational and emotional needs of women who may be hesitant or unable to visit clinics [37]. However, accessing doula support can be challenging for some participants due to service cost, lack of awareness about available resources, and implicit bias. Specifically, Black community-based doulas, as patient advocates, help address these disparities through offering services that are not provided by conventional doulas [38]. However, Black doulas themselves may encounter obstacles, such as racism, when providing care and interacting with the health care system [38]. To address these barriers, community-based organizations can integrate doula service information, making it readily available and accessible to women in need [39]. In addition, raising awareness about the benefits of doula support and addressing any misconceptions or concerns surrounding their involvement during the pandemic is essential. Furthermore, expanding insurance coverage for doula services and ensuring hospital policies allow their presence in delivery room can further enhance access to doula support.

Institutional-level barriers also influence women’s access to desired maternal care experiences during COVID-19. COVID-19 control policies implemented by hospitals and clinics, such as mandatory COVID-19 testing and visitor restrictions, directly impact the quality of care received by women [40]. These findings complement existing studies that have examined the impact of changing COVID-19 prevention and control policies in maternal care [40]. In the event of a pandemic or other health emergency, health care organizations need to implement flexible policies that can quickly respond to changing situations while ensuring quality patient care. Moreover, to ensure equitable treatment and reduce disparities in care during such public health crises, it is essential to develop comprehensive guidelines and a robust monitoring system. These guidelines should be patient-centered, ensure continuity of care, address issues at different levels, and promote clear communication. The monitoring system should be designed to quickly identify and address any inequities in care delivery, ensuring that all women receive consistent access to maternal care services. This dual focus on adaptability and a patient-centered approach is critical to meeting the challenges of rapid policy change and maintaining a supportive and compassionate environment for maternal health. Furthermore, the frequent changes in policy and protocol, combined with the impact of staff shortages, underscore the importance of preparedness and timely response from MCPs [41,42]. Studies have shown that the health care provider shortage during the pandemic has strained the health care system and increased the workload of MCPs, further resulting in compromised quality of care [43,44]. The combination of rapidly evolving guidelines, limited staffing resources, and increased patient demands further challenges the ability of MCPs to provide optimal services [43,45]. Amidst these challenges, telehealth has developed as an effective tool in facilitating access to maternal care services [46]. It offers convenience and flexibility, which can be particularly beneficial for women with busy schedules, transportation limitations, or childcare responsibilities. Equitable access to telehealth is necessary to ensure that all women can benefit from these services without exacerbating existing disparities [47,48]. Socioeconomic factors and disparities in digital literacy may limit the ability of some women, especially those from low-income or underserved communities, to effectively engage with telehealth services [49]. By recognizing these impacts, equitable access to resources should be prioritized, along with the improvement of telehealth services and the tailoring of policies to address the unique needs and preferences of diverse populations.

AA and Hispanic women face significant health disparities in maternal care, leading to higher rates of complications and adverse outcomes during pregnancy and childbirth [5,9]. To address these disparities and improve the utilization of maternal care services among these populations, targeted interventions are necessary. First, leveraging the resources of community health workers and doulas, while empowering social support groups and local communities, is crucial. Community health workers and doulas play a vital role in providing support, guidance, and advocacy to AA and Hispanic women. By integrating community health workers and doulas into the maternal care services, women can receive the comprehensive support, assistance, and individualized care required to improve their health outcomes. This collaborative approach helps to distribute the workload more evenly, thereby reducing the burden on MCPs and improving the overall quality of care. To mitigate implicit bias among Black doulas and MCPs, training programs and workshops can be implemented to enhance cultural competence and sensitivity [50,51]. In addition, increased collaboration between community health workers, doulas, and MCPs can establish a resilient care system, ensuring continuous care even during periods of workforce shortages. Second, strengthening telehealth as an integral component of maternal care is also paramount. By expanding telehealth capabilities and making it more accessible, women can overcome barriers to timely access of important health care information and ultimately reducing health disparities. However, the implementation of telehealth and the efficacy and quality of telehealth may be influenced by many existing infrastructure conditions [52–54]. More studies are needed to explore the relevant policy and capacity building strategies to avoid generating new types of disparities.

This study has several limitations. First, the study was conducted solely in one state (i.e., SC), which may not reflect the experiences of pregnant and postpartum women in other southern states, potentially limiting the generalizability of our findings. Second, the study did not include AA and Hispanic women who did not come to the clinics to use the maternal care services, limiting the understanding of their unique challenges in accessing maternal care services during COVID-19. Third, the absence of a comparison group of White women limits our ability to compare the experiences in maternal care utilization across different racial and ethnic groups. Fourth, our Hispanic participants primarily consisted of immigrants, and their experiences with the health care system may differ from those of US-born Hispanics.

Future research should address these limitations and further investigate the impact of the COVID-19 pandemic on women who do not have access to clinic-based maternal care services. In addition, the experiences of people of different races and ethnicities, socioeconomic backgrounds, and geographic locations should be explored to better understand the disruptions of public health crises on maternal care utilization and provision. Given the diversity within the Hispanic population and considering factors such as their acculturation and language proficiency, further research could provide a more nuanced understanding of the unique challenges they face in accessing maternal care services. In addition to addressing these limitations in future research, we can remove barriers to maternal care and improve health outcomes for every mother and child through program implementation and policy improvements. Understanding the barriers and challenges that prevent the use of maternal care services is important for developing strategies for consistent maternal care. Furthermore, evaluating the effectiveness of various interventions aimed at improving the quality and accessibility of maternal care services during public health crises could help mitigate health disparities and ensure equitable access to care for underserved populations.

## Conclusion

The COVID-19 pandemic has significantly disrupted the health care system, especially affecting the utilization and provision of maternal care services. Addressing these challenges needs a comprehensive approach that integrates community partner resources and employs appropriate telehealth strategies. In addition, it is important to prioritize mental health services and social support to alleviate the stress and anxiety experienced by pregnant and postpartum women. Early identification of substance use should incorporate assessments of recent stressful life events and the availability of support. Health education groups or classes could be beneficial to assist pregnant and postpartum women in maintaining healthy lifestyles, managing stress, coping with negative emotions, and seeking assistance. Tailoring culturally adapted interventions to meet the specific needs of AA and Hispanic pregnant and postpartum women can help reduce the burden of COVID-19 on them. Furthermore, providing adequate resources and support for MCPs will enhance service quality and improve women’s maternal health outcomes.

## Supporting information

S1 Table Interview guide for women. (DOCX)

S2 Table Interview guide for maternal care providers. (DOCX)

S1 File (DOCX)
