# Supplementary material for: Maternal care utilization and provision during the COVID-19 pandemic: Voices from minoritized pregnant and postpartum women and maternal care providers in Deep South
Source: PLoS One. 2024 Apr 29;19(4):e0300424. doi: 10.1371/journal.pone.0300424 (PMC11057746; doi:10.1371/journal.pone.0300424)
Supplement: S1 Table — (DOCX) [file pone.0300424.s001.docx]

# Supporting information

**S1 Table. Interview Guide for Women.**

| **Domain** | **Example Questions** |
| --- | --- |
| Sociodemographic background | - How old are you? - What is your current level of education? - What about your current relationship status? (e.g., single, married, unmarried partnership, divorced, etc.) - How many children do you have? How old are they? - Where are you living since the recent pregnancy? (which county?) - Did you have health insurance at the time of your pregnancy? What is it? Do you have Medicaid? - Where were you born? (country’s name) How long have you been in US? (if you are not born in US) |
| Stressors in the COVID-19 pandemic | - Is there anything that made you feel stressed or anxious during the pregnancy? - Is there anything that makes you feel stressed and anxious during the postpartum period? - How would you like to describe your psychological conditions during the pregnancy? Did you have any problems sleeping? How was your mood or feelings? - How would you like to describe your psychological conditions after giving birth? Did you have any problems sleeping? How was your mood or feelings? |
| Experience with prenatal, intrapartum, and postpartum care | Pregnancy and prenatal   - Did you visit any doctors during the pregnancy? If yes, how did you know where to go? If not, why? - Were you visited by community health workers? - Could you describe the clinic where you received care? - Could you describe the doctors who saw you? (Hispanic: did they speak Spanish?) - Can you share your experience of getting care during your pregnancy? - If you could change anything about the services you received during the pregnancy, what would you change? - Did you have any complications during that pregnancy? What is it? How did the doctors deal with it? Did they explain it to you?   Intrapartum   - Where did you have your baby? Would you like share the birthing experience? - Do you believe that the COVID-19 affected the services you received at delivery? - If there was any unexpected change related to your labor (e.g., natural birth, cesarean delivery, induced labor etc.), did your provider explain to you the reasons for the change? - If you could change anything about the services you received during delivery, what would you change?   Postpartum   - Did you have a postpartum visit within 3 months after birth? Did you have any postpartum health problems? If so, how did your doctor say what you should do? If you did not visit any doctor after birth, why did you not go? - Did the clinic give you any information on how to keep you and your baby healthy after delivery? - Do you believe that COVID-19 affected the quality of service you received? If so, how? - If you could change anything about the services you received during the postpartum visit, what would you change? |
| Perceptions of maternal care providers | - How were your feelings about the providers who took care of you during pregnancy, at delivery, after delivery? - Did you trust them? Did you feel they treated you with respect? |
| Challenges in healthcare seeking | - Did you miss any appointments or clinic visits during your pregnancy and postpartum period? Any reasons for these missing visits? - Did you have any concerns in seeking professional help or visiting health clinics? For example, did you worry about medical bills, health insurance? Or the attitudes of the doctors and nurses, front desk staff? - When seeking out health care, was there anything that made you feel uncomfortable, or feel that you were treated unfairly? - If you could advise the clinics or hospitals to make changes to improve their service, what would you recommend they change? |
| Social support | - Did any of your family members, friends, co-workers help care for you and your children (including the newborn baby) during the pandemic? What did they do? - If they did not provide much service or help, what would you have liked them to do? |
| Needs and recommendations for future healthcare | - If there could be a special program designed to assist pregnant and postpartum women like you throughout the COVID-19 pandemic, what would you like to see from that program? - Do you have any suggestions for how that program could best support African American (Hispanic) women? |
