# Supplementary material for: Maternal care utilization and provision during the COVID-19 pandemic: Voices from minoritized pregnant and postpartum women and maternal care providers in Deep South
Source: PLoS One. 2024 Apr 29;19(4):e0300424. doi: 10.1371/journal.pone.0300424 (PMC11057746; doi:10.1371/journal.pone.0300424)
Supplement: S2 Table — (DOCX) [file pone.0300424.s002.docx]

# Supporting information

**S2 Table. Interview Guide for Maternal Care Providers.**

| **Domain** | **Example Questions** |
| --- | --- |
| Sociodemographic background | - What is your age? - How long have you been working here? - What is your current job title? What do you do in your daily work? - I know that some of your clients are ethnic minority, could you estimate the racial composition? |
| Impacts of COVID-19 on care provision | - What are the challenges providing maternal care (e.g., prenatal care, intrapartum care, and postnatal care) in the communities you serve during the pandemic? Could you provide some examples? How did you deal with these challenges? - Are there any differences in labor and delivery in your facilities during the COVID-19 compared with pre-pandemic? Could you provide some examples? What do you think of these changes? How did you deal with these differences? - How did COVID-19 change the policies and practices in the department/unit where you work? - Do you think the changes have affected the quality of care for women? How? - Do you think the changes have affected maternal health outcomes? How? - Are there any changes in practice or procedure that you think should be continued beyond COVID-19? If so, which? |
| Stressors and challenges of their clients | - Based on your observations, what are the specific challenges that your patients have experienced in the pandemic? - What has made them stressed and anxious? |
| Clients’ mental health conditions | - How would you like to describe your clients’ psychological conditions during the pregnancy in general? Did they complain about any problems related to mental health? - What about their psychological conditions after giving birth? |
| Perceptions and views on health disparities caused by structural factors | - What do you think about the health disparity in maternal health outcomes in South Carolina? In your opinion, what are the main factors that contribute to the disparities? - How did the COVID-19 pandemic affect these factors? - Do you have any suggestions to reduce health disparity in maternal health in South Carolina? |
| Needs/recommendations for future healthcare | - If there was an intervention, or a program, dedicated to addressing the maternal health of African American (Hispanic) women throughout the COVID-19 pandemic, what would you like to see from that program? - Do you have any suggestions for how that program could best support African American (Hispanic) women? |
