# Supplementary material for: Maternal care utilization and provision during the COVID-19 pandemic: Voices from minoritized pregnant and postpartum women and maternal care providers in Deep South
Source: PLoS One. 2024 Apr 29;19(4):e0300424. doi: 10.1371/journal.pone.0300424 (PMC11057746; doi:10.1371/journal.pone.0300424)
Supplement: S1 File — (DOCX) [file pone.0300424.s003.docx]

**Supplemental data project:** Data for the publication of *Maternal care utilization and provision during the COVID-19 pandemic: Voices from minoritized pregnant and postpartum women and maternal care providers in Deep South*.

**Project name:** *Multilevel Determinants of Racial/Ethnic Disparities in Maternal Morbidity and Mortality in the Context of the COVID-19 Pandemic*

**Qualitative data type:** semi-structured interview

|  | **African American Participants** | **Hispanic Participants** | **Maternal Care Providers** |
| --- | --- | --- | --- |
| intrapersonal level: fears and stress during the pandemic | “If I saw her name [MCP’s name], I'd be like, y'all can't change it? And most of the time, she was the only one available because everybody else had surgery. So that didn't make me not want to go. But it didn't put me in the right mindset before I showed up. It wasn't like I was excited, but it's kind of hard to be excited when she's gonna ruin it for me.” (AA 7)  “I am worried about that, when I go to these doctor's appointments, am I gonna bring something back to her? ... I was very cautious.” (AA 13)  “But all in all, it was stressful too, like going to the doctor with COVID going on, and having to wear a mask, and you're just trying to stay safe.” (AA 15)  “The COVID had me stressed out 'cause I didn't know how it would affect a pregnant person, and so I talked to my doctor, and they advised me to get the vaccines and everything.” (AA 19)  "When we got COVID, I was extra scared because of not knowing how it affected her [the baby]. Our doctor, she really, I'm not gonna say brushed me off, but I call her to tell her that, I was just diagnosed with COVID-19. What do I do? And her exact words were: ‘Nothing. We don't treat pregnant mothers differently than we do any other person that has COVID’. And that kind of scared me because I'm just like, well, how would COVID affect my baby? It was no education behind nothing.” (AA 4)  "Yes, very stressed. Very stressful. Being a full-time mom, being a full-time worker, being a wife, during a pandemic is not easy.” (AA 4) | “During pregnancy, one is very sensitive and if you add all the issues with the pandemic, everything is complicated.” (Hispanic 6) | “It's also part of the patients as well not wanting to come in because they don't want to be exposed to COVID either, that maybe people don't come in soon enough for things that they needed to come in for, like high blood pressure issues, and just various things.” (MCP 3)  “Even if you're happy about being pregnant, whereas some people are still ambivalent, it's still stressful.” (MCP 5)  "I do have a lot of people that just freely admit to smoking marijuana, even pregnant ... I'm like, ‘let's talk about that’.” (MCP 5)  “... probably marijuana use. We ask in terms of tobacco and alcohol. We have very few patients that will admit to alcohol and tobacco. A lot of our patients, it's marijuana and [I’ve] probably seen an increase in that just them saying, it helps me relax.” (MCP 2)  "They said to me ... ‘I've done that for the nausea in pregnancy’. [It] was one of the things that they've always said is, ... ‘I find that, that helps when I'm nauseous’. I'm like, well, there are safer things that you can do for nausea than that.” (MCP 1)  “I'm like, are you still smoking marijuana? [patient says] ‘Yeah’, I'm just like, Okay, well, we'll do the third trimester drug screen. They're like, okay, but they don't seem to be bothered by that. But they disclose it. I'm very impressed that patients disclose more of that now since COVID.” (MCP 1) |
| Interpersonal level: family and social network | “I didn't even bring [infant's name] with me [to doctor appointment], even though they told me over the phone, I could. But I didn't bring her for personal reasons. Because of COVID. 'Cause I didn't want her to be exposed and be around her sister. But I think I think maybe if we weren't in a pandemic, she probably would have been with me.” (AA 13)  “If I had a doctor's appointment and dad wasn't around, she [participant’s mother] would pick us up and take us.” (AA 17)  “My mom had him the whole time I was in the hospital.” (AA 18)  “I guess yeah, sometimes his great grandma would keep him, but it would be like if I had an appointment.” (AA 2)  “... sister groups most definitely ... You think you're going through a lot with your pregnancy, and [to know] there's someone out there shares the same fears and things, and I feel like um sister groups like community groups and things of that sort can kind of lay off the pressure of pregnancy. Like you're not in this by yourself.” (AA 13)  “Like even a support group for, even for people who just need someone to talk to.” (AA 3)  “… probably like how the [name of program] that they have there in Columbia. I think they should probably have something like that with um parents here too. At least somebody to walk me through the entire pregnancy ... just ‘cause you get WIC [Women, Infants and Children Nutrition program], WIC is not enough.” (AA 16) | “Yes, when I had to go to appointments, a friend helped me or pick up my girl from school.” (Hispanic 6) | “If a spouse gets COVID, that's happened a number of times the spouse got COVID and had to isolate. So now you've got a new baby. Her spouse is in the house, can't help you at all. There's other little children. And now you're taking care of a new baby, and you just have to see, it's just a mess.” (MCP 5)  “I do know, at [hospital name] as I'm sure that you've heard, the centering was shut down, and it is having a lot of trouble being picked back up.” (MCP 9]  “Centering Pregnancy model is really a great model, because...you don't know what you don't know. And so, if you've got 20 women in a room, and they're doing this, they a lot of times will feel power in having people like them, they're pregnant, or they're all African American, or they're Latina, or they're a combination, but there's more pregnant people in the room, than there are doctors, or nurses or whatever. So, that dynamic, it shifts, it really does. And they ask questions, and one of them will ask [a] question that the other one didn't even know to ask, and she's like, ‘Oh’, and they learn from each other. And I think, I think that it's really powerful, and COVID just kind of destroyed that in so many ways that for those centering groups.” (MCP 5)  “I think I would like to see more community efforts to kind of recover that, like centering that we had, like from all the things I learned about centering, it sounded great. Like it was improving outcomes. It was improving like the mom's mental health, and then they started to have like, centering for parenting.” (MCP 8)  “But they're not getting a two-hour diabetes and pregnancy class, they're not getting group education. And I think there's benefits of the group setting as well and knowing that you're not alone, and they, they're not getting the same education and care simply because of the fact that there's the language barrier. So I would like to see classes for the Spanish speaking women.” (MCP 9) |
| Community level: doulas and community health workers | “'Cause they kept me straight. They were like, ‘Listen, if you don't do this, this is gonna happen. If you do this, this'll happen’. They were straight up forward. Letting me know everything, always gave me the information I needed. Um, if they couldn't talk to me, my mom and fiancé were always on call. So, they would let them know everything. So, they were on top of everything.” (AA 19)  "Her care was amazing. Through the third trimester and throughout my labor, I was an anxiety wreck 'cause she was coming a month early ... I'll never forget, I was crying. I was like, I've never even got my hair braided. 'Cause was supposed to go get my hair braided the next day ... So I was just crying like mid-contraction. I was like, I didn't even get my hair braided. So she braided my hair. She braided my hair in like two little French braids. And that just made me just feel just, it was like a detail like that, that I know, I wouldn't have gotten at a hospital. She was like, it's okay, I'll braid your hair. So she just braided my hair. So that way when I gave birth, my hair wasn't going everywhere looking crazy. It was immaculate care.” (AA 6)  “Everybody was wearing masks. So I couldn’t really gauge the excitement. I guess that kind of threw me ‘cause, my doula was in a mask. Everybody was in a mask. The only people that weren’t in masks were obviously me and my husband. So I think the, the social cues of, is she coming? ... I can't really see what's happening right now.” (AA 6)  "As a Black woman, giving birth in the US and ... giving birth at home, I think a program like that will be very beneficial. If people knew that these services are not expensive. I went into the impression when I started this, that this was gonna be so expensive, I could never afford this, I would have to just settle for the hospital. Because that's just all we were taught. And that's all I knew. Nobody told me anything ... And we just assume not trying to be funny, but only white people can afford them. And that's not true at all.” (AA 6) | “She supported me a lot. With visits and by phone. Each time she visited me, she helped me because everything was more difficult due to the pandemic. She facilitated everything for me.” (Hispanic 20)  “They gave me fliers with all the care. The reasons why we needed to go back to the hospital.” (Hispanic 19)  “I would have liked to have been offered doula services. I know what a doula is and how that service could have benefitted me because of this support for the mother, especially for me that I didn't have any support during the delivery with me. I know moms from other races that they get offered doula services and to me they didn't offer it and didn't even tell me they had that service.” (Hispanic 3) | “Um also came to find out after working with a group of doulas that there were doulas that were being turned away and the doulas that were being turned away, were black doulas. So, we also have the implicit bias going on, because people are making decisions about who can be there and who can't be there. And that didn't used to be an issue when whoever could be present.” (MCP 3) |
| Institutional level: new policy and regulations implemented in health facilities, and uncertainty associated with changing policies | “The only thing that was different was not having my husband come to any of the appointments. So, um I went into everything alone. Like honestly, the only appointment he went to was when I delivered and that that was it. That's the only appointment he was able to come to so just feeling I wasn't, I wouldn't say I was scared because I've been through many doctors' appointments, but it was just that extra sense of comfort when he was there with my, our son. I just wasn't able to have him there with our daughter which was, it was scary man.” (AA 4)  “When I found out I was pregnant, they weren't allowing anybody in the birthing room besides the mother, the nurse and the doctor. So, no family, no anything. And that made me anxious because I'm a first-time mom, I've never given birth before. I was like, of course I want my husband there, at least my mom, but I would take my husband, but it was in the beginning my gynecologist was like, No, you can't have anybody. And that just immediately turned me off. So, I started looking at other things.” (AA 6)  “I felt like my rights were being, excuse me, my rights were being violated as a patient because you have the right to moral support. So, I just felt like I understood this was for safety purposes, but it was a little bit too strict, just not having that one person.” (AA 11)  “I was concerned going in, 'cause they have to COVID test you to go in, which they didn't COVID test my husband, which was so stupid. I was like, that doesn't make any sense to me. But anyway, so I had to get a COVID test.” (AA 10)  “... that visitation policy and have to be subjected to the COVID testing. That was very, it was a straight violation of me. I just didn't like it. The mandatory testing.” (AA 11)  “So now that covid is out, whether you vaccinated or not. I feel like they do get a little nervous closed up in there. So they're not taking the time that they usually do, or they get you in and out.” (AA 7)  “Hmm. I mean, probably because I think due to COVID I don't know if hospitals were always short staffed in that manner. But I know that there was a boom of healthcare workers quitting after COVID happened. So they were extremely short staffed. It did play a part in ... me not getting the aftercare that I was supposed to get after delivery.” (AA 18) | “Yes, I was panicked about not knowing about the pandemic and that we couldn't do the prenatal visits normally. I had to go in by myself because they didn't allow my husband to be with me.” (Hispanic 20)  “COVID affected because now there are too many rules. Everybody has to wear a face mask and I couldn't go to the appointment with someone else. I had a different idea of how this was going to go.” (Hispanic 17)  “It was very confusing. They took a long time to get appointments. They didn't allow anyone to be with you. When one has a complicated pregnancy, one needs support.” (Hispanic 2) | “But the policy changes that, that wouldn't allow partners to come or any flexibility ... or the screening practices and ... all of the questions around who might be positive or exposed, it turned a lot of women away from appointments and care, like if they showed up with their children, they were turned away, or if they had a temp, or if they had a sniffle, they might be turned away and not cared for.” (MCP 9)  “[patients would ask] why do I have to get a babysitter to come to prenatal care, when I used to be able to bring my kids with me to the visits? And a lot of them can't afford to do that. So, we see a lot of missed visits because of that, they didn't have a babysitter.” (MCP 1)  “We used to not obsess over patients, they'd get a cold, or they'd get a sniffle, or they'd get a flu, we would just provide care to them and take care of them. Now we're like, Oh, you gotta go get a COVID test before we can even see you. I think it's really affected how we provide care to patients. Everything is like everything's rule out COVID before you see the patient.” (MCP 1)  “I do know that when I was a nurse, and supporting a patient with an epidural, not every nurse, but I feel like the good nurses really supported their patients, you touch them, you're close to them. They have to lean up around you and hold on to you to get their back in the right position. But if you're all masked up and afraid to COVID, you're just not touching people very much, you're not putting your hands on them. And getting right there with them to provide that hands on support and care.” (MCP 9)  "Um, I honestly think the thing that led to the quality of care issues, and I do think in some ways the quality was diminished... I think that that was probably due to the implicit bias that we had. And the fear that providers and nursing had, that they were going to get the virus. So we, I'm gonna use we as me included, even though I tried not to do this, but we would do everything we could not to see the patient if she was in that 10 day window of having COVID. And I think sometimes that was done. Those decisions were made out of concern, slash fear, for [the] provider team more than it was for the patient themselves. And we probably didn't see some patients that we should have seen or that we would normally would have seen.” (MCP 7)  "And then also, if there's a concern that somebody's positive, or they are positive, they're getting less one on one care, because you have to get in your full garb and dressed up so they're not getting the support that they were getting before. So now...they don't have their labor support people with them and now they don't have their nurses with them either. So, I think that's a huge difference.” (MCP 3)  " ... and then it affects staffing, when patients come into the office sick, we have staff members that then get sick with COVID. And then they're out, which then affects how much care we can provide.” (MCP 1)  "There's less tolerance, I guess of, of people who are wanting things, like for birth, a lot of people have their birth plans, and they'd like to have things a certain way. And I think the nursing staff is a little shorter than they used to be. And part of that is the burnout that's happening for them. So, they're not as flexible and, and that definitely affects patient care.” (MCP 3)  “And then in the hospital ... when we started to recognize that pregnant patients fared very poorly with COVID ... trying to a lot of times, doing it on the fly, trying to extrapolate information from other spheres of medicine to apply to a pregnant patient and breastfeeding patients, and so forth, that that was challenging. There's a lot of on your feet thinking and extrapolating from other parts of medicine.” (MCP 5)  “Cause you had to take everything off. At the time PPE was not as, it was scarce, I mean, you were kind of worried like, am I gonna have all the supplies? I need to actually care for somebody without making myself sick.” (MCP 4)  “There was the whole thing about like, we had a shortage of PPE for a while ... And then it was like, they kind of kept relaxing their requirements when we didn't have enough PPE and saying, like, ‘oh, you actually can wear the same N95 all day’ ... they only said that, because we didn't have enough.” (MCP 8)  “At the very beginning with donning and doffing with PPE that was initially supervised, there was like always a watcher to watch and make sure you did it properly. So, that of course changed when we didn't have enough people to do that. And then I think it's masking is obviously the biggest one, having really strict rules about masking, who is masked what having to wear a mask all the time, and that is constantly under flux.” (MCP 6)  “It did open up the opportunities for virtual visits ... But it took away that personal touch, and the ability, particularly in pregnancy, when you really need to lay your hands on a mom's belly, listen to the heartbeat, look at the patient. You can only go so far with a virtual visit. So, it decreased that personal touch and, and I think decreased our ability to truly physically assess the patient as well as we would like to in a normal situation.” (MCP 7)  “A lot of the technology that we had just wasn't accessible to those patients. So it's all well and good to say we'll do a video visit and have you check your own like blood sugars at home, but if you can't access a glucometer or glucose strips because you can't afford them, or you have spotty Wi-Fi or you live somewhere rural where you don't have Wi-Fi, then that's not really a possibility. So, I just felt like they didn't have the same access to tools that other people were using to try and overcome pandemic stuff.” (MCP 6)  “One of the good things that has come out of COVID is the expectation that people will be able to use telemedicine in some capacity, or that we will have some sort of like, drive through some sort of component of healthcare. And that has been actually very good because those one-on-one interactions for short periods of times actually do serve a great purpose ... if you need to have a telehealth visit, whereas before it was a complete novelty.” (MCP 5)  "I think one of the challenges, is ... the changing policies, when patients ask, ... when I go to have my baby, how many people can come? Or can it be two people in the room now? And or can they switch out, and I'm like, it changes so much that I don't know how to, to counsel them to give them clear expectations.” (MCP 9)  "I think patients are more bothered by it. In terms of they feel like we're treating them differently. And I mean I guess we are, but it's not their fault. I mean it's not them per se. ... I think patients may say that ... because it may not be as personal when you have a gown and a mask and a face shield and gloves and all that on, but I don't think the overall care has decreased or changed.” (MCP 2) |
